# Impaired rapid error monitoring but intact error signaling following rostral anterior cingulate cortex lesions in humans

**DOI:** 10.3389/fnhum.2015.00339

**Published:** 2015-06-17

**Authors:** Martin E. Maier, Francesco Di Gregorio, Teresa Muricchio, Giuseppe Di Pellegrino

**Affiliations:** ^1^Department of Psychology, Catholic University of Eichstätt-IngolstadtEichstätt, Germany; ^2^Centro Studi e Ricerche in Neuroscienze Cognitive, Polo Scientifico-Didattico di Cesena, Alma Mater Studiorum Università di BolognaCesena, Italy; ^3^Dipartimento di Psicologia, Alma Mater Studiorum Università di BolognaBologna, Italy

**Keywords:** performance monitoring, error-related negativity (Ne/ERN), error detection, event-related potentials, brain injury

## Abstract

Detecting one’s own errors and appropriately correcting behavior are crucial for efficient goal-directed performance. A correlate of rapid evaluation of behavioral outcomes is the error-related negativity (Ne/ERN) which emerges at the time of the erroneous response over frontal brain areas. However, whether the error monitoring system’s ability to distinguish between errors and correct responses at this early time point is a necessary precondition for the subsequent emergence of error awareness remains unclear. The present study investigated this question using error-related brain activity and vocal error signaling responses in seven human patients with lesions in the rostral anterior cingulate cortex (rACC) and adjoining ventromedial prefrontal cortex, while they performed a flanker task. The difference between errors and correct responses was severely attenuated in these patients indicating impaired rapid error monitong, but they showed no impairment in error signaling. However, impaired rapid error monitoring coincided with a failure to increase response accuracy on trials following errors. These results demonstrate that the error monitoring system’s ability to distinguish between errors and correct responses at the time of the response is crucial for adaptive post-error adjustments, but not a necessary precondition for error awareness.

## Introduction

Error detection and correction are critical for optimizing goal-directed behavior. An electrophysiological correlate of rapid error monitoring is the error negativity (Ne; Falkenstein et al., 1990) or error-related negativity (ERN; Gehring et al., 1993) emerging over fronto-central scalp areas following errors. The Ne/ERN is most likely generated in the dorsal anterior cingulate cortex (dACC) located in the medial prefrontal cortex (e.g., Debener et al., 2005), which is believed to signal the need for behavioral adjustments aimed at optimizing performance following errors and other unfavorable outcomes (Ridderinkhof et al., 2004). Accordingly, the Ne/ERN amplitude has been found to be positively related to the amount of adaptive behavioral adjustments following errors such as post-error slowing (Debener et al., 2005).

However, despite extensive research, it is not yet clear how the Ne/ERN and the subsequent emergence of conscious error awareness are related. Most extant studies have investigated this relationship by comparing Ne/ERN amplitudes for aware and unaware errors (see, Wessel, 2012, for a review). Some studies have found larger Ne/ERN amplitudes for aware than for unaware errors (e.g., Scheffers and Coles, 2000; Maier et al., 2008; Steinhauser and Yeung, 2010; Hewig et al., 2011; Wessel et al., 2011), while other studies have not reported such an effect (e.g., Nieuwenhuis et al., 2001; Endrass et al., 2007; O’Connell et al., 2007; Shalgi et al., 2009; Hughes and Yeung, 2011).

Several factors could account for these inconsistent findings. For instance, larger Ne/ERNs for aware relative to unaware errors have often been found in tasks, which produce errors due to data limitations such as tasks using stimulus masking (Maier et al., 2008), stimulus degradation (Scheffers and Coles, 2000), discriminations of perceptually similar stimuli (Steinhauser and Yeung, 2010) or difficult and complex tasks (Hewig et al., 2011). Under such conditions, the representation of the correct response is impaired on many trials, which leads to both attenuated Ne/ERNs and impaired error signaling (Scheffers and Coles, 2000; Hewig et al., 2011). Thus, smaller Ne/ERNs for unaware errors under conditions, which frequently elicit errors due to data limitations, cannot be taken as evidence that the rapid error evaluation process associated with the Ne/ERN provides the basis for the later emergence of error awareness. Other authors have argued that differences in the precise methods of measuring error awareness can influence whether Ne/ERN differences between aware and unaware errors can be found or not (e.g., Wessel, 2012). For instance, differences in the Ne/ERN between aware and unaware errors are more often seen when participants indicate on each trial whether they think they have responded correctly or incorrectly (Wessel et al., 2011), or rate how confident they are of having committed an error (Shalgi and Deouell, 2012; Navarro-Cebrian et al., 2013), compared to simple error reporting solely on error trials (e.g., Nieuwenhuis et al., 2001; Hughes and Yeung, 2011).

Thus, comparing Ne/ERN amplitudes for aware and unaware errors presents a number of problems. To circumvent this, one can investigate whether neurological patients with impairments of the Ne/ERN are still capable of signaling their errors. Patients with lesions of the rostral anterior cingulate cortex (rACC) and adjoining ventromedial prefrontal cortex defined as Brodman areas 24a-c, 25, 32, and 33 (e.g., Bush et al., 2000) have been previously shown to display severely attenuated Ne/ERN amplitudes (Stemmer et al., 2004; Turken and Swick, 2008). The neural generator of the Ne/ERN is most often localized to the dACC (e.g., Debener et al., 2005). However, attenuated Ne/ERN amplitudes in patients with lesions of the rACC suggest that the mechanism generating the Ne/ERN is dependent on input from an intact rACC, possibly due to this brain region’s role in evaluating the negative consequences of errors (Turken and Swick, 2008). In addition, spared error awareness has been reported in patients with lesions spanning both the dACC and the rACC (Modirrousta and Fellows, 2008). Thus, taken together, the findings from these two lines of inquiry seem to indicate that an intact Ne/ERN is not necessary for the emergence of error awareness.

However, for drawing a strong conclusion, it is crucial to demonstrate reduced Ne/ERN and intact error awareness in the same sample of patients. Three out of four of the patients examined by Modirrousta and Fellows (2008) with intact error awareness had unilateral lesions mostly restricted to the left dACC/rACC. Thus, it is possible that spared portions of the dACC/rACC in the intact hemisphere afforded intact error awareness, and that these spared portions would also have generated a sizeable Ne/ERN in these patients. Indeed, a recent study found entirely intact Ne/ERN in two patients with unilateral dACC lesions (Løvstad et al., 2012; but see Swick and Turken, 2002). Unfortunately, Modirrousta and Fellows (2008) did not include EEG data to clarify whether intact error awareness correlated with an intact Ne/ERN. Conversely, the two previous studies that demonstrated reduced Ne/ERN with rACC lesions did not report any measures of error awareness (Stemmer et al., 2004; Turken and Swick, 2008).

Therefore, we investigated both error-related brain activity and error awareness in seven patients with lesions of the rACC. Their performance was compared to age-matched neurologically healthy controls and also with neurological patients with lesions outside the frontal lobes. Participants performed a flanker task (Eriksen and Eriksen, 1974) and were required to indicate on each trial whether they thought that they had responded correctly or had committed an error (Wessel et al., 2011). We hypothesized that rACC patients would display impaired rapid error monitoring activity but unimpaired error awareness. This would demonstrate that the rACC is crucial for rapid error monitoring but not for error awareness. Additionally, we measured adaptive post-error adjustments of behavior in the form of post-error slowing and post-error accuracy increase as it remains unclear whether such adjustments are associated with rapid error monitoring, with error awareness or with both (see, Danielmeier and Ullsperger, 2011; Wessel, 2012).

## Materials and Methods

### Participants

Three groups of male individuals participated in the study: (a) a group of patients with lesions centered on the rACC and the adjoining ventromedial PFC (rACC group, *n* = 7, mean age = 54.7 years, SE = 3.80 years); (b) a brain damaged control group of patients with lesions outside of the the rACC and the adjoining ventromedial PFC (BDC group, *n* = 7, mean age = 57.6 years, SE = 5.04 years); and (c) a group of neurologically healthy age-matched control subjects (HC group, *n* = 7, mean age = 48.0 years, SE = 3.33 years).

Brain-damaged patients were recruited from the Center for Studies and Research in Cognitive Neuroscience in Cesena, Italy. They were selected on the basis of lesion location by inspection of computed tomography (CT) or structural magnetic resonance imaging (MRI) scans. At the time of testing, all patients were more than a year post onset, were not taking psychoactive drugs and were free of any other diagnosis likely to affect cognitive control processes such as significant psychiatric disease, alcohol misuse, or a history of cerebrovascular disease. Participants in the HC group were matched to the rACC group for age and education and recruited through local advertising. Participants gave written informed consent before participating in the study and all procedures were carried out according to the Declaration of Helsinki (International Committee of Medical Journal Editors, 1991) and approved by the Ethical Committee of the University of Bologna.

Table 1 shows demographic and clinical data, as well as subjects’ scores in the Mini-Mental Status Examination score (MMSE; Folstein et al., 1983). As shown by two-sided independent-samples *t*-tests, demographic data did not significantly differ between the rACC group and the HC group, all *p*s < 0.208, nor did they differ between the rACC group and the BDC group, all *p*s < 0.659. rACC patients were additionally screened for cognitive impairment using a standardized neuropsychological test battery, and performance was compared to large sample normative data following the methodology proposed by Capitani (see, Capitani, 1997), in which raw scores are first adjusted for age, sex, and schooling, and then transformed into standardized scores (named Equivalent Scores, ES) on an ordinal scale ranging from 0–4 (ES = 0 represents pathological performance, and ES = 4 represents performance higher than the median). Specifically, the neuropsychological test battery assessed selective attention (Attentional Matrices; Spinnler and Tognoni, 1987), reasoning (Raven’s Colored Progressive Matrices; Basso et al., 1987; Verbal Judgment Task; Spinnler and Tognoni, 1987), verbal fluency (Novelli et al., 1986), verbal and visuospatial short-term memory (Digit and Corsi Span; Orsini et al., 1987), and verbal long-term memory (Babcock Prose Recall Test; Spinnler and Tognoni, 1987). All but one of the rACC patients showed ES in the normal range on all measures (see Table 2), which is in accordance with previous reports (Bechara et al., 1998, 2000, 2005).

**Table 1:** **Demographic, clinical and lesion data of patients with lesions of the rostral anterior cingulate cortex (rACC Group), of brain damaged control patients (BDC Group), and demographic and clinical data of healthy controls (HC Group)**.

Group	Age (Years)	Education (Years)	Lesion volume (cc)	MMSE score
rACC	54.7 (3.80)	11.0 (1.76)	48.4 cc (10.5 cc)	28.1 (0.78)
HC	48.0 (3.33)	13.7 (1.30)	–	29.3 (0.36)
BDC	57.6 (5.04)	10.7 (2.03)	43.9 cc (8.40 cc)	28.0 (0.93)

*Standard errors of means are given in parenthesis*.

**Table 2:** **Neuropsychological data of patients with lesions of the rostral anterior cingulate cortex**.

Test	ES score	n.v.
Attentional Matrices	3.29 (0.360)	7
Digit Span	3.29 (0.565)	7
Corsi Span	3.14 (0.553)	7
Verbal Fluency Phonological Cue	2.00 (0.378)	6
Verbal Fluency Semantic Cue	3.00 (0.436)	6
Raven Colored Matrices	3.14 (0.459)	7
Prose Memory	1.71 (0.459)	7
Verbal Judgment Task	2.57 (0.297)	7

*Mean Equivalent Scores (ES) ranging from 0–4 with values of 0 representing pathological and values of 4 representing above median performance and the number of patients with normal values (n.v.) are shown for each neuropsychological test. Standard errors of means are given in parenthesis*.

**Figure 1:**
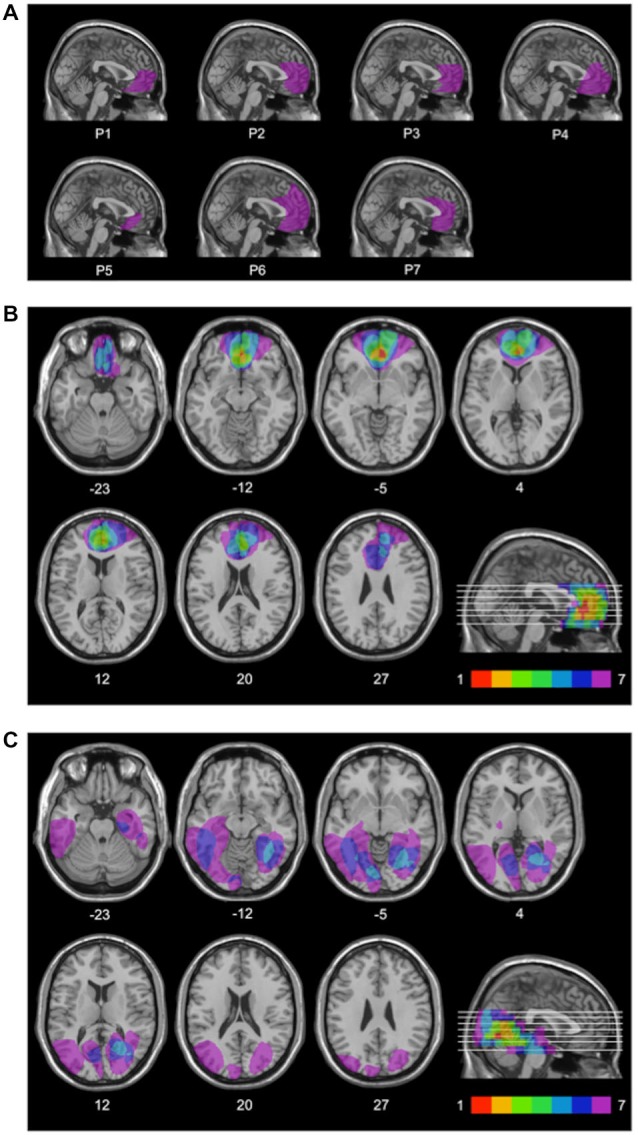
**Lesion locations**. Mesial view of the standard Montreal Neurological Institute brain showing the individual lesions in the rACC group **(A)**, lesion overlap in the rACC group **(B)**, and lesion overlap in the BDC group **(C)** each projected on the same seven axial slices and on the mesial view of the standard Montreal Neurological Institute brain. In **(B)** and **(C)**, levels of axial slices are marked by white horizontal lines on the mesial views of the brain, and *z*-coordinates of each axial slice are given. Color bars indicate the number of overlapping lesions. In each axial slice, the left hemisphere is located on the left side. In the rACC group, maximal lesion overlap occurs in the rostral portion of the anterior cingulate cortex and adjoining ventromedial prefrontal cortex.

For each patient, lesion extent and location were documented by using the most recent clinical CT or MRI scan. Lesions were manually drawn by a neurologist with experience in image analysis onto the T1-weighted template MRI scan from the Montreal Neurological Institute provided by the MRIcro software (Rorden and Brett, 2000). This scan is normalized to Talairach space and is a popular template for normalization in functional brain imaging. Superimposing each patient’s lesion onto the standard brain allowed us to estimate the total brain lesion volume in cubic centimeters (cc). The same MRIcro software was used to overlay individual brain lesions. Figure 1A shows the individual brain lesions and Figure 1B the lesion overlap in the rACC group. In this group, lesions were restricted to the rostral portion of the medial surface of the frontal lobe, and were the result of ruptured aneurysms of the anterior communicating artery. The Brodmann’s areas with most extensive damage were areas 10, 11, 24, 25, and 32, i.e., the rACC and adjoining ventromedial PFC regions. Six patients in the rACC group had bilateral lesions, and one patient had a unilateral right-sided lesion.

Lesion overlap in the BDC group is shown in Figure 1C. In this group, lesions only affected cerebral cortex outside the frontal lobes. All BDC patients had lesions in the occipital lobe, with four of them also having lesions in the temporal lobe, and three of them also having lesions in the parieto-temporo-occipital junction. Lesions of patients in the BDC group were the result of ischemic (*n* = 5) or hemorrhagic (*n* = 2) stroke. None of the BDC patients had lesions in areas 10, 11, 24, 25, or 32, i.e., the anterior cingulate cortex and adjoining ventromedial PFC. In the BDC group, all patients had unilateral (*n* = 4 left-sided, *n* = 3 right-sided) lesions. There was no significant difference in lesion volume between rACC patients (48.4 cc, SE = 10.5 cc) and BDC patients (43.9 cc, SE = 8.40 cc), *t*_(12)_ = 0.335, *p* = 0.744, *η*_p_^2^ = 0.009.

### Apparatus

A PC running Presentation software (Neurobehavioral Systems, Albany, CA, USA) controlled stimulus presentation and response registration. Stimuli were presented on a 17-inch color monitor at a viewing distance of 80 cm.

### Task and Procedure

**Figure 2:**
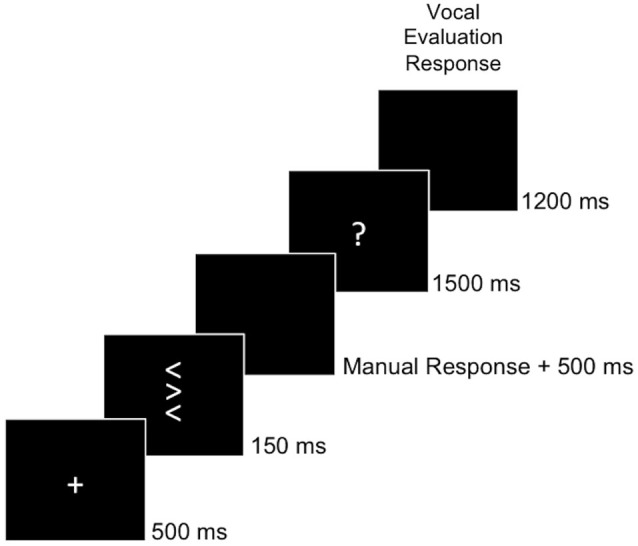
**Behavioral Task**. The task was to classify the central target arrow with respect to its direction by a manual response. After the manual response, a question mark prompted participants to evaluate their own behavior by saying “giusto” (engl. correct) if they thought having responded correctly or “sbagliato” (engl. wrong) if they thought having responded erroneously.

Participants were seated in a dimly lit room in front of a computer screen. They performed a flanker task (Eriksen and Eriksen, 1974) in which they were asked to respond by key-press to the direction of a central target arrowhead (left or right) while ignoring two identical distractor arrowheads presented above and below the target. On 50% of the trials, distractor arrowheads pointed in the same direction as the target (congruent stimuli), while on the other 50% of the trials, distractor arrowheads pointed in the opposite direction of the target (incongruent stimuli). Each arrowhead subtended a visual angle of 0.64° * 0.57° (height * width), and the whole stimulus array subtended a visual angle of 2.22° height. All stimuli were presented in the screen center. An example trial is depicted in Figure 2. On each trial, a white fixation cross was presented for 500 ms. Then, the stimulus appeared for 150 ms followed by a black screen. After the participants’ response, the screen remained black for 500 ms, during which Ne/ERN and Pe could be measured. After this interval, a secondary error awareness task followed. To this end, a white question mark appeared for 1500 ms. Participants were instructed to indicate whether they thought they had responded correctly or erroneously to the stimulus by saying the Italian words “giusto” (English: “correct”) or “sbagliato” (English: “wrong”), respectively, after the appearance of the question mark. The experimenter manually recorded the participants’ vocal response on each trial for later analyses of error awareness. After the question mark, the screen turned black for 1200 ms before the next trial started. If a second response occurred after the initial response (e.g., a spontaneous error correction) during any of the response windows, then this response was recorded as well and the respective interval (first black screen, question mark or second black screen) was restarted. Participants completed eight blocks of 64 trials, each of which was preceded by three randomly drawn practice trials. This amounted to 512 test trials for the analyses. Before the start of the experiment, participants performed one block of 32 trials without the error awareness task, and one block of 64 trials in conjunction with the error awareness task for practice. In addition, participants underwent a practice session on a day preceding the experiment proper. In the practice session, participants first performed five blocks of 32 trials each without the secondary error awareness task. After each of these blocks, participants were instructed to respond more quickly, if their error rate on incongruent trials of the preceding block was below 20%. This was done to ensure a sufficient number of error trials for the analyses. After practicing the flanker task alone, participants performed a mean of five blocks of 64 trials of the flanker task in conjunction with the error awareness task to practice maintaining the required response speed while evaluating their own performance by responding vocally after the appearance of the question mark on each trial.

### Psychophysiological Recording

During the test session, the electroencephalogram (EEG) was recorded with Ag/AgCl electrodes (Fast’n Easy-Electrodes, Easycap, Herrsching, Germany) from 27 electrode sites (Fp1, F3, F7, FC1, FC5, C3, CP1, CP5, P3, P7, O1, AFz, Fz, FCz, Cz, Pz, Fp2, F4, F8, FC2, FC6, C4, CP2, CP6, P4, P8, O2) and from the right mastoid. The left mastoid was used as reference, and the ground electrode was placed on the right cheek. The electrooculogram (EOG) was recorded from above and below the left eye and from the outer canthi of both eyes. EEG and EOG were recorded with a band-pass filter of 0.01–100 Hz, amplified by a BrainAmp DC amplifier (Brain Products, Gilching, Germany), digitized at a sampling rate of 1000 Hz, and re-sampled to 500 Hz offline.

### Data Analyses

#### Task Performance

**Figure 3:**
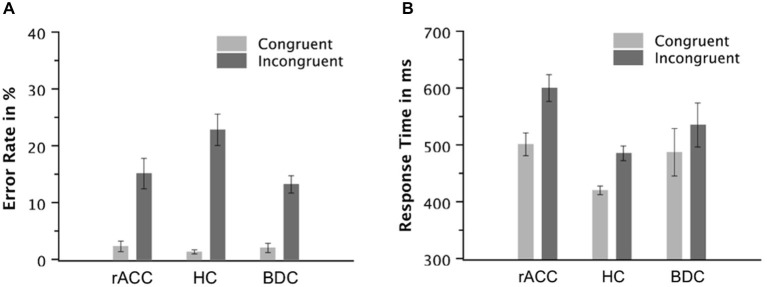
**Task Performance**. Error rates in % **(A)** and response times of correct responses in milliseconds **(B)** on congruent and incongruent trials in the flanker task in patients with lesions of the rostral anterior cingulate cortex (rACC Group), in healthy controls (HC Group), and in patients with brain lesions outside the frontal lobes (BDC Group). ms, milliseconds; Error bars represent standard errors of the mean.

RT was defined as the time between stimulus onset and the button press. If an error was followed by a correct response, this was registered as correct error correction. Inappropriate second responses were defined as repetitions of the error on error trials, repetitions of the correct response on correct trials or execution of the erroneous response after an initial correct response. Trials with RTs deviating more than four standard deviations from the condition mean were excluded from RT analyses (<1% of all trials). Frequency data were arcsine-transformed for statistical testing (Winer et al., 1991). RT of correct responses and error rates were analyzed by two-way mixed-model ANOVAs with repeated measurement on the between-subjects variable group (rACC, HC, BDC) and the within-subjects variable congruency (congruent, incongruent). Between-group planned comparisons were performed on the difference between incongruent and congruent trials using two-tailed independent-samples *t*-tests. RT of incongruent errors was analyzed by a one-way mixed model ANOVA with repeated measurement on the between-subjects variable group (rACC, HC, BDC). For the analyses of error RT, only data from incongruent trials were considered, as there were very few errors on congruent trials (see Figure 3).

#### ERP Data

EEG data were analyzed using EEGLAB v13.0.1 (Delorme and Makeig, 2004) and custom routines written in MatLab R2012b (The Mathworks, Natick, MA, USA). ERP data were re-referenced off-line to the average of both mastoids (Luck, 2005), and filtered with a 1–20 Hz pass-band. For the response-locked averages, epochs of 200 ms before and 600 ms after the response were extracted from the continuous EEG and baseline-corrected using a 150 ms to 50 ms pre-response window. This window was chosen to avoid losing error-related activity due to the divergence of error and correct waveforms already before the response with the response-locked baseline (see e.g., Riesel et al., 2013). Notably, the pattern of statistical results remained the same, when response-locked ERPs were analyzed using the average voltage in a time window of 100 ms preceding the stimulus as baseline. For the stimulus-locked averages, epochs of 200 ms before and 800 ms after stimulus onset were extracted and baseline-corrected using a 100 ms pre-stimulus onset window. Epochs were excluded using the pop_autorej function in EEGLAB v13.0.1, which first excludes trials with voltage fluctuations larger than 1000 μV, and then excludes trials with data values outside five standard deviations using an iterative algorithm. The mean percentage of excluded trials was 5.33% (SE = 0.79%) for the response-locked averages and 5.21% (SE = 0.58%) for the stimulus-locked averages. To correct remaining artifacts, the data were then subjected to a temporal ICA (Jutten and Herault, 1991; Makeig et al., 1996) using the infomax algorithm (Bell and Sejnowski, 1995). The resulting component matrix was screened for independent components (ICs) representing stereotyped artifact activity, such as horizontal (saccades) and vertical (blinks) eye movements, and muscle artifacts. This was done using a multistep correlational template-matching process as implemented in CORRMAP v1.02 (Viola et al., 2009). Topographies of ICs labeled as artifacts by the CORRMAP procedure were visually inspected and then calculated out of the data using inverse matrix multiplication.

Rapid error monitoring was measured by the mean voltage in an interval of −10 ms to 90 ms relative to the response (Steinhauser and Yeung, 2010) and analyzed at fronto-central electrode FCz. Additionally, we investigated the later error positivity (Pe; Falkenstein et al., 2000), which is seen 200 ms following errors. The Pe is usually larger for aware than for unaware errors and therefore attributed to conscious error detection (see, e.g., Overbeek et al., 2005). Thus, we expected that the Pe is unimpaired in rACC patients. The Pe was quantified as the mean voltage in a 100 ms time window centered around the most positive peak of the error—correct difference waveform in a time window of 200 ms to 400 ms following the response and analyzed at the parietal midline electrode Pz. This method was used to control for variations in the peak of the Pe between participants. The electrodes for the analyses of both components were chosen based on the scalp topographies in the HC group (see Figure 6). The time windows for the analyses of both components were chosen based on previous literature (Steinhauser and Yeung, 2010). Both rapid error monitoring and Pe were analyzed by two-way mixed-model ANOVAs with repeated measurement on the between-subjects variable group (rACC, HC, BDC) and the within-subjects variable response (correct, error). Between-group planned comparisons were performed on the difference between error and correct trials using two-tailed *t*-tests. Rapid error monitoring has been measured by the difference between errors and correct responses (henceforward ΔNe/ERN) in the peri-response time window in many previous studies (e.g., Gehring and Knight, 2000; Yeung et al., 2004; Turken and Swick, 2008; Steinhauser and Yeung, 2010; Wessel et al., 2014), because it is thought to capture the error monitoring system’s ability to distinguish errors from correct trials and to signal the need for appropriate behavioral post-error adjustments. However, in order to additionally investigate whether differences in rapid error monitoring between groups could be attributed to differences in the Ne/ERN on error trials or to differences in the negativity on correct trials (correct-related negativity, CRN), planned contrasts using two-tailed *t*-tests between groups were also calculated separately for error and correct trials. Only data from incongruent trials were considered for the analyses of response-locked ERP data, as there were very few errors on congruent trials (see Figure 3).

Finally, to investigate whether rACC lesions have a general detrimental effect on ERPs, we investigated the P300 in the stimulus-locked averages. To quantify the P300, the mean voltage in a 200 ms time window centered around the most positive peak in a time window of 400–700 ms post stimulus-onset was used. This method was used to control for variations in the peak of the P300 between participants (see also, Dundon et al., in press). The P300 was analyzed by a two-way mixed-model ANOVA with repeated measurement on the between-subjects variable group (rACC, HC, BDC) and the within-subjects variable congruency (congruent, incongruent). Between-group planned comparisons were performed using two-tailed *t*-tests.

#### Post-Error Behavior

To analyze error awareness, the proportion of correct incongruent responses indicated by the participants as errors relative to all incongruent trials with correct responses (false alarms), and the proportion of incongruent error trials indicated by the participants as errors relative to all incongruent trials with erroneous responses (hits) were used to calculate parameter free A′ values according to signal detection theory (Stanislaw and Todorov, 1999). A′ values were analyzed by a one-way between-subjects ANOVA on the variable group (rACC, HC, BDC). Frequencies of error trials with error corrections relative to all error trials were analyzed by a two-way mixed-model ANOVA with repeated measurement on the between-subjects variable group (rACC, HC, BDC) and the within-subjects variable correction type (correct error correction, false second response). Finally, two measures of post-error behavioral adjustments were considered. First, post-error slowing was quantified as the difference in RT of correct responses immediately preceding and immediately following errors (Dutilh et al., 2012). Second, post-error increase of accuracy was quantified as the difference in error rate on trials following correct responses and on trials following errors. Post-error slowing and post-error increase of accuracy were analyzed by two-way mixed-model ANOVAs with repeated measurement on the between-subjects variable group (rACC, HC, BDC) and the within-subjects variable response on trial n-1 (correct, error). Between-group planned comparisons were performed on the difference between error and correct trials using two-tailed *t*-tests. For the analyses of behavioral adjustments, trials on which a second response was registered after the initial response were excluded, as they can distort measures of behavioral adjustments (Rabbitt, 1968; Rabbitt and Rodgers, 1977). One patient in the rACC group corrected all errors. Data from this patient were therefore excluded from the analyses of post-error behavioral adjustments. Notably, error corrections can affect the Ne/ERN (Fiehler et al., 2005). However, all Ne/ERN results remained the same when the participant correcting all errors was excluded from the analyses. For the analyses of error awareness and error correction frequencies, only data from incongruent trials were considered, as there were very few errors on congruent trials (see Figure 3).

## Results

### Task Performance

The behavioral data are depicted in Figure 3. The ANOVA on the error rates revealed main effects of group, *F*_(2,18)_ = 4.42, *p* = 0.024, *η*_p_^2^ = 0.339, and congruency, *F*_(1,18)_ = 74.9, *p* < 0.001, *η*_p_^2^ = 0.806. Furthermore, the ANOVA revealed an interaction of the variables group and congruency, *F*_(2,18)_ = 4.80, *p* = 0.021, *η*_p_^2^ = 0.348. Planned comparisons showed that the congruency effect calculated as the difference between error rates on incongruent and congruent trials tended to be larger in the HC group (21.5%, SE = 2.73%) than the rACC group (12.7%, SE = 2.03%, *t*_(12)_ = 2.08, *p* = 0.060, *η*_p_^2^ = 0.265) and the BDC group (11.2%, SE = 2.01%, *t*_(12)_ = 3.09, *p* < 0.009, *η*_p_^2^ = 0.443). However, the congruency effect was comparable between the rACC group and the BDC group (*p* < 0.501, *η*_p_^2^ = 0.039). Further planned comparisons showed that the larger congruency effect in the error rates in the HC group was mainly driven by the error rate on incongruent trials. Error rates on congruent trials were comparable across groups, all *p*s < 0.337, *η*_p_^2^ s < 0.077. However, error rates on incongruent trials tended to be larger in the HC group than the rACC group, *t*_(12)_ = 2.04, *p* = 0.064, *η*_p_^2^ = 0.258, and were larger in the HC group compared to the BDC group, *t*_(12)_ = 3.15, *p* = 0.008, *η*_p_^2^ = 0.452. Error rates on incongruent trials were comparable between the rACC group and the BDC group, *p* = 0.494, *η*_p_^2^ = 0.040.

The ANOVA on RT of correct responses revealed a main effect of congruency, *F*_(1,18)_ = 320, *p* < 0.001, *η*_p_^2^ = 0.947, denoting that RT was higher on incongruent trials (540 ms, SE = 18.2 ms) than on congruent trials (469 ms, SE = 16.9 ms). Furthermore, the ANOVA revealed an interaction of the variables group and congruency, *F*_(2,18)_ = 14.4, *p* < 0.001, *η*_p_^2^ = 0.615. Planned comparisons showed that the congruency effect calculated as the difference in RT between incongruent and congruent trials was larger in rACC group (99 ms, SE = 9.0 ms) than in both the HC group (66 ms, SE = 5.7 ms, *t*_(12)_ = 3.11, *p* = 0.009, *η*_p_^2^ = 0.446) and the BDC group (48 ms, SE = 5.1 ms, *t*_(12)_ = 4.94, *p* < 0.001, *η*_p_^2^ = 0.670). The difference in RT between incongruent and congruent trials was also larger in HC group than in the BDC group (*t*_(12)_ = 2.33, *p* = 0.038, *η*_p_^2^ = 0.311). Further planned comparisons showed that RTs on congruent trials were faster in the HC group than in the rACC group, *t*_(12)_ = 3.82, *p* = 0.002, *η*_p_^2^ = 0.548, but comparable between the HC group and the BDC group, *p* = 0.244, *η*_p_^2^ = 0.176, and between the rACC group and the BDC group, *p* = 0.771, *η*_p_^2^ = 0.007. RTs on incongruent trials were faster in the HC group than in the rACC group, *t*_(12)_ = 4.28, *p* = 0.001, *η*_p_^2^ = 0.604, but comparable between the HC group and the BDC group, *p* = 0.244, *η*_p_^2^ = 0.111, and between the rACC group and the BDC group, *p* = 0.178, *η*_p_^2^ = 0.146.

The ANOVA on incongruent error RT did not reveal a significant effect (*p* < 0.284, *η*_p_^2^ < 0.130) suggesting that incongruent error RTs were similar across groups (rACC group: 458 ms, SE = 22.3 ms; HC group: 410 ms, SE = 6.53 ms; BDC group: 496 ms, SE = 59.3 ms).

In sum, all groups of participants responded more slowly and less accurately on incongruent than on congruent trials. However, error rates on incongruent trials were larger in the HC group than in the other two groups. By contrast, for RT, the congruency effect was largest in the rACC group, intermediate in the HC group, and smallest in the BDC group.

### ERP Data

#### Rapid Error Monitoring Activity

**Figure 4:**
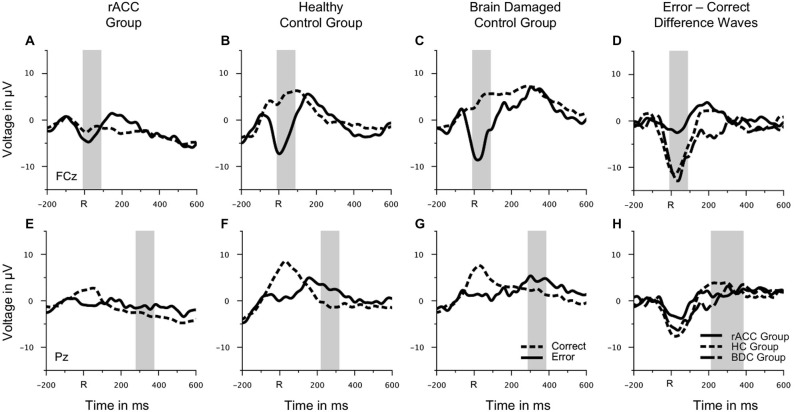
**Response-locked ERP data**. Grand average waveforms for correct and erroneous responses at electrode FCz **(A–C)** and at electrode Pz **(E–G)** as well as response-locked grandaverage difference waveforms (correct—error) at electrode FCz **(D)** and at electrode Pz **(H)** in patients with lesions of the rostral anterior cingulate cortex (rACC Group), in healthy controls (HC Group), and in patients with brain lesions outside the frontal lobes (BDC Group). R = time point of the button press; μV = microvolt; shaded areas correspond to the time windows used for statistical analyses.

Grand average response-locked ERP waveforms for correct and error trials at electrode FCz are shown in Figures 4A–C. Waveforms for error trials were more negative than waveforms for correct trials in the typical Ne/ERN time window around the button press in all three groups. Waveforms of this ΔNe/ERN at electrode FCz are shown in Figure 4D. The ΔNe/ERN seemed much smaller in the rACC group than in the other two groups. Scalp topographies of the mean voltage difference between error and correct trials in a time window of −10 to 90 ms relative to the response are shown in the upper row of Figure 4, where clear frontocentral maxima corresponding to a typical Ne/ERN topography are visible in both the HC and the BDC groups. By contrast, in the rACC group, no clear ΔNe/ERN maximum is identifiable. Statistical analyses confirmed these impressions. The ANOVA on the ΔNe/ERN revealed a main effect of response, *F*_(1,18)_ = 45.9, *p* < 0.001, *η*_p_^2^ = 0.718, denoting more negative waveforms on error trials (−4.73 μV, SE = 1.15 μV) than on correct trials (2.59 μV, SE = 1.22 μV). Furthermore, and crucially for the present purpose, an interaction of group and response, *F*_(2,18)_ = 6.62, *p* = 0.007, *η*_p_^2^ = 0.424 was observed. Planned comparisons revealed that the difference between error and correct trials in the rACC group (−1.79 μV, SE = 0.997 μV) was smaller than the difference between error and correct trials in both the HC group (−9.62 μV, SE = 1.33 μV; *t*_(12)_ = 3.84, *p* < 0.002, *η*_p_^2^ = 0.552) and the BDC group (−10.6 μV, SE = 2.79 μV; *t*_(12)_ = 2.97, *p* < 0.012, *η*_p_^2^ = 0.423). The difference between error and correct trials was comparable in the HC group and in the BDC group, *p* = 0.698, *η*_p_^2^ = 0.001. Thus, rapid error monitoring activity quantified as the difference between error and correct trials (see also, e.g., Gehring and Knight, 2000; Yeung et al., 2004; Turken and Swick, 2008; Steinhauser and Yeung, 2010; Wessel et al., 2014) was clearly impaired in the rACC group compared to both the HC group and the BDC group.

**Figure 5:**
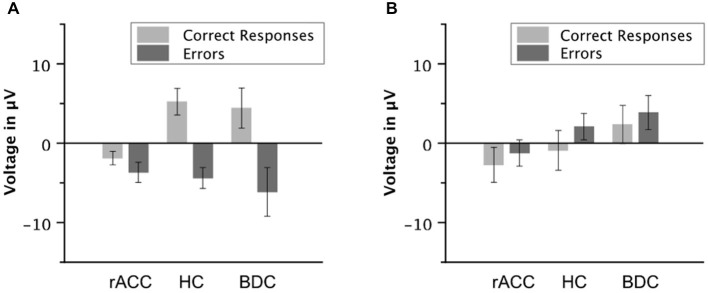
**Ne/ERN and Pe amplitudes**. Average mean amplitudes of correct and error waveforms in the Ne/ERN time window **(A)** and in the Pe time window **(B)** in patients with lesions of the rostral anterior cingulate cortex (rACC Group), in healthy controls (HC Group), and in patients with brain lesions outside the frontal lobes (BDC Group). μV = microvolt; error bars represent standard errors of the mean.

To investigate, whether this effect was due to an attenuation of the negativity on error trials or due to an enhancement of the negative deflection on correct trials, the mean voltage in the Ne/ERN time window was also compared separately for correct and error trials (e.g., Gehring and Knight, 2000). The resulting amplitudes are shown in Figure 5A. The negativity in the Ne/ERN time window on error trials was not different across groups (rACC group: −3.67 μV, SE = 1.27 μV; HC group: −4.39 μV, SE = 1.32 μV; BDC group: −6.14 μV, SE = 3.07 μV), all *p*s < 0.742, *η*_p_^2^ s < 0.044. However, the negativity on correct trials was significantly enhanced in the rACC group (−1.89 μV, SE = 0.845 μV) compared to both the HC group (5.23 μV, SE = 1.68 μV; *t*_(12)_ = 3.79, *p* < 0.003, *η*_p_^2^ = 0.545) and the BDC group (4.43 μV, SE = 2.53 μV; *t*_(12)_ = 2.37, *p* < 0.036, *η*_p_^2^ = 0.318). This shows that the reduction of the ΔNe/ERN in the rACC group was more likely due to an enhancement of the negativity on correct trials than due to a reduction of the negativity on error trials.

As different task performances are believed to affect the ΔNe/ERN, for instance through differences in post-error response conflict (Yeung et al., 2004) or through differences in error expectancy (Holroyd and Coles, 2002; but see, Maier et al., 2012), it is important to consider differences in task performance between the groups. The differences in task performance between the rACC group and the HC group (higher error rate on incongruent trials in the HC relative to the rACC group, but longer RT on correct incongruent trials in the rACC than in the HC group) might be best explained by a difference in the balance between speed and accuracy with a higher emphasis on speed than on accuracy in the HC group. Note that such liberal responding may lead to smaller ΔNe/ERN amplitudes (Gehring et al., 1993; but see, Steinhauser and Yeung, 2012). Thus, if differences in ΔNe/ERN amplitudes between the rACC group and the HC group were driven by differences in task performance, one would expect larger ΔNe/ERN amplitudes in the rACC group than in the HC group. However, we observed the opposite, i.e., larger ΔNe/ERN amplitudes for HC than for rACC patients. This makes it unlikely that differences in the ΔNe/ERN between the HC group and the rACC group were due to differences in RT between those groups. Moreover, error rates were very similar between the rACC group and the BDC group. Thus, effects of error expectancy can be excluded as a factor driving differences between the rACC and the BDC group. The only difference in task performance between the rACC and the BDC group was a larger congruency effect on correct RT in the rACC group driven by numerically slower RT on incongruent correct trials in the rACC than in the BDC group. To exclude the possibility that these differences in RT between the groups are responsible for effects on the ΔNe/ERN, we repeated the ΔNe/ERN analyses on a subset of trials matched for RT between the rACC group and the BDC group. To this end, we used the following algorithm (Maier and Steinhauser, 2013): first, each rACC patient was paired with the BDC patient with the smallest overall RT difference without replacement. Second, for correct and error trials separately, the participant with fewer trials in the respective condition was selected (either rACC or BDC). Third, a trial from the respective condition in this participant was randomly drawn without replacement. Forth, from the other participant, the trial providing the closest match to the RT of the firstly drawn trial was selected without replacement and assigned to the RT-matched sample. These steps were repeated until all trials from the participant with the smaller trial number in the respective condition were drawn.

A mixed-model ANOVA with repeated measurement with the between-subjects variable group (rACC, BDC) and the within-subjects variable response (correct, error) on RT in the subset of RT-matched trials yielded a main effect of response, *F*_(1,12)_ = 9.00, *p* = 0.011, *η*_p_^2^ = 0.429, with faster RT on error trials (567 ms) than on correct trials (482 ms). No further effects emerged (all *p*s < 0.814, *η*_p_^2^ < 0.005). Importantly, after RT-matching, RT did not differ significantly any more between the rACC group (520 ms, SE = 32.3 ms) and the BDC group (529 ms, SE = 31.4 ms), *t*_(12)_ = 0.173, *p* = 0.865, *η*_p_^2^ = 0.003.

A mixed-model repeated measurements ANOVA with the between subjects variable group (rACC, BDC) and the within subjects variable response (correct, error) on ΔNe/ERN amplitudes on the subset of RT-matched trials yielded a main effect of response, *F*_(1,12)_ = 22.1, *p* < 0.001, *η*_p_^2^ = 0.648, denoting more negative waveforms on error trials (−5.11 μV, SE = 1.70 μV) than on correct trials (1.55 μV, SE = 1.50 μV). Crucially, the interaction between group and response was significant as well, *F*_(1,12)_ = 8.21, *p* = 0.014, *η*_p_^2^ = 0.407, reflecting a larger difference between error and correct trials in the BDC group (−10.7 μV, SE = 2.51 μV) than in the rACC group (−2.59 μV, SE = 1.22 μV. Thus, it is unlikely that differences in RT are responsible for the observed ΔNe/ERN differences between the rACC group and the BDC group.

#### Pe

**Figure 6:**
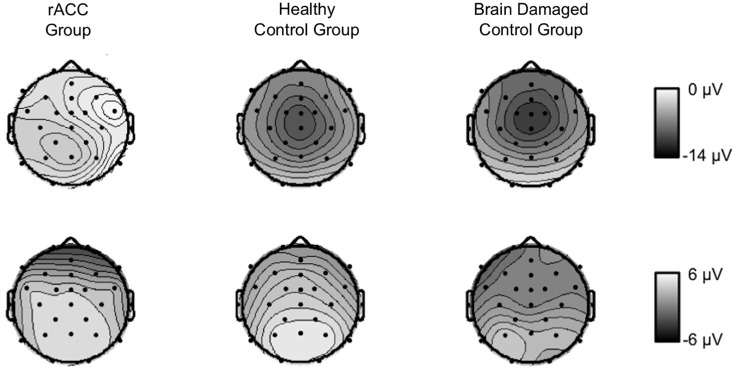
**Scalp topographies of response-locked ERP data**. Grand average scalp topographies of the difference between error and correct waveforms in the Ne/ERN time window (−10 to 90 ms relative to the response) and in the Pe time window (100 ms centered around the most positive peak of the error—correct difference waveform in a time window of 200 ms to 400 ms following the response) in patients with lesions of the rostral anterior cingulate cortex (rACC Group), in healthy controls (HC Group), and in patients with brain lesions outside the frontal lobes (BDC Group). μV = microvolt.

Grand average response-locked ERP waveforms for correct and error trials at electrode Pz are shown in Figures 4E–G, amplitudes are shown in Figure 5B. In all three groups of participants, the waveforms of error trials were more positive than those of correct trials in the time window of the Pe from around 200–400 ms after the response. The Pe was somewhat smaller compared to previous reports, which is possibly due to preparation of the vocal error signaling response in the Pe time window. As is evident from the scalp topographies (Figure 6), the Pe had typical parietal maxima in all experimental groups (cf. Steinhauser and Yeung, 2010). Therefore, and consistent the previous Pe literature (see Overbeek et al., 2005, for a review), we chose to analyze the Pe on channel Pz for all three groups. The ANOVA on the Pe revealed a significant main effect of response, *F*_(1,18)_ = 6.25, *p* = 0.022, *η*_p_^2^ = 0.303 indicating larger amplitudes on error trials (0.870 μV, SE = 0.940 μV) than on correct trials (−1.20 μV, SE = 1.02 μV). No other effects reached significance (all *p*s < 0.229, *η*_p_^2^ < 0.151). Importantly, there was no interaction between group and response, *p* = 0.687, *η*_p_^2^ = 0.041 suggesting that the Pe was comparable between the groups. An ANOVA with the between subjects variable group (rACC, HC, BDC) on the peak latencies did not reveal any significant differences between the rACC group (328 ms SE = 29.4 ms post-response), the HC group (273 ms SE = 27.3 ms post-response), and the BDC group (331 ms SE = 28.4 ms post-response), *p* = 0.285, *η*_p_^2^ = 0.130.

#### P300

**Figure 7:**
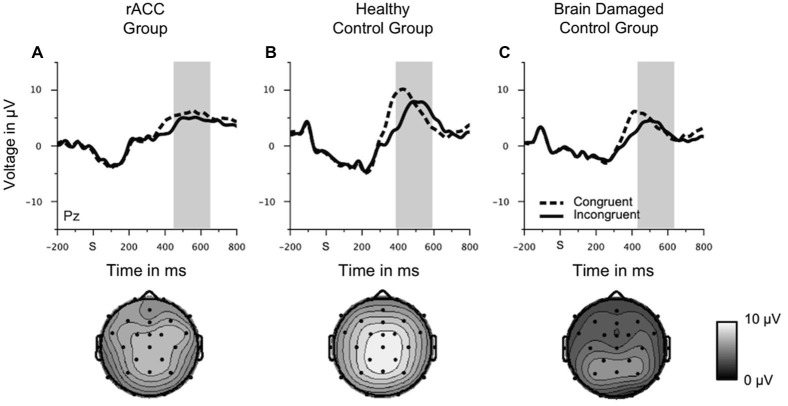
**Stimulus-locked ERP data**. Grand average waveforms for correct congruent and correct incongruent trials at electrode Pz as well scalp topographies of the mean voltage in a 200 ms window centered around the individual most positive voltage peak in a time window of 400 ms to 700 ms following the stimulus in patients with lesions of the rostral anterior cingulate cortex (rACC Group, **A**), in healthy controls (HC Group, **B**), and in patients with brain lesions outside the frontal lobes (BDC Group, **C**). S = time point of stimulus onset; μV = microvolt; shaded areas correspond to the time windows used for statistical analyses.

Grand-average stimulus-locked waveforms and topographies of the P300 are shown in Figure 7. Although the parietally distributed P300 seemed larger in the HC group than in both the rACC group and the BDC group, it seemed comparable between the two patient groups. These impressions were confirmed by statistical analyses. The ANOVA on P300 amplitudes revealed a main effect of congruency, *F*_(1,18)_ = 11.2, *p* = 0.004, *η*_p_^2^ = 0.382 denoting a larger P300 for congruent (3.84 μV, SE = 0.734 μV) than for incongruent stimuli (2.98 μV, SE = 0.707 μV). No other effects emerged, all *p*s < 0.423, *η*_p_^2^ s = 0.091. This shows that ERPs were not generally blunted in the patient groups. An ANOVA with the between-subjects variable group (rACC, HC, BDC) and the within-subjects variable congruency (congruent, incongruent) on the peak latencies did not reveal any significant effects, all *p*s < 0.394, *η*_p_^2^ s < 0.041. Importantly, it did not reveal differences in the peak latencies between the rACC group (545 ms SE = 26.6 ms post stimulus-onset), the HC group (486 ms SE = 17.9 ms post-response), and the BDC group (532 ms SE = 31.9 ms post-response), *p* = 0.495, *η*_p_^2^ = 0.075.

### Post-error Behavior

#### Error Awareness

**Figure 8:**
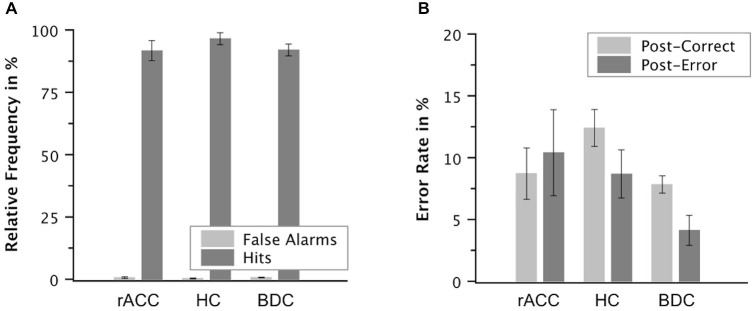
**Post-error behavior**. Mean relative frequencies of correct and error trials followed by the vocal response “Wrong!” (false alarms and hits, respectively, **A**) and mean error rate in % on trials following correct responses and on trials following errors in patients with lesions of the rostral anterior cingulate cortex (rACC Group), in healthy controls (HC Group), and in patients with brain lesions outside the frontal lobes (BDC Group, **B**). Error bars represent standard errors of the mean.

Proportions of false alarms on correct incongruent trials relative to all correct incongruent trials and proportions of hits on incongruent error trials relative to all incongruent error trials are shown in Figure 8. While relative frequencies of false alarms seemed smaller than relative frequencies of hits in all three groups of participants, there seemed to be no group differences in error awareness. Mean A′ values were high (98.0, SE = 0.520) indicating good error awareness. The ANOVA on A′ values did not reveal a main effect of group, *F*_(2,18)_ = 0.795, *p* = 0.467, *η*_p_^2^ = 0.083. Also when compared directly, A′ values were comparable between the rACC group and the control groups (rACC: 97.7, SE = 1.12 vs. HC: 99.0, SE = 0.670, *t*_(12)_ = 1.01, *p* = 0.333, *η*_p_^2^ < 0.078; rACC vs. BDC: 97.7, SE = 0.650, *t*_(12)_ = 0.032, *p* = 0.982, *η*_p_^2^ < 0.001). Importantly, effect sizes were consistently very low (all *η*_p_^2^ s < 0.083), and extremely low (*η*_p_^2^ s < 0.001) for the comparison of A^′^ values between the rACC and BDC groups.

#### Error Corrections

Although error corrections were not explicitly instructed, participants corrected a substantial number of errors. The ANOVA on the proportions of correct and incorrect corrections on incongruent error trials relative to all incongruent error trials revealed a main effect of correction type, *F*_(1,18)_ = 28.4, *p* < 0.001, *η*_p_^2^ = 0.612 with more correct error corrections (30.4%, SE = 5.12%) than false error corrections (0.636%, SE = 0.419%). No further effects reached significance (all *p*s < 0.423, *η*_p_^2^ s < 0.091). Importantly, the frequencies of error corrections were comparable between the three experimental groups (rACC: 20.4%, SE = 8.51%; HC: 12.0%, SE = 4.79%; BDC: 14.2%, SE = 3.70%), *p* = 0.423, *η*_p_^2^ = 0.091). These results remained qualitatively the same, when the rACC patient who corrected all errors was excluded (all *p*s < 0.609, *η*_p_^2^ s < 0.057).

#### Post-error Behavioral Adjustments

The ANOVA on the error rates revealed an interaction of group and response, *F*_(2,17)_ = 5.27, *p* = 0.017, *η*_p_^2^ = 0.383. Planned comparisons showed that post-error increase of accuracy was significantly smaller in the rACC group (−1.83%, SE = 2.70%) than in both the HC group (3.70%, SE = 1.14%), *t*_(11)_ = 2.76, *p* = 0.019, *η*_p_^2^ = 0.409, and the BDC group (3.51%, SE = 1.32%), *t*_(11)_ = 2.21, *p* = 0.049), *η*_p_^2^ = 0.308. By contrast, post-error increase of accuracy did not differ between the HC group and the BDC group, *p* < 0.327, *η*_p_^2^ = 0.081.

Post-error slowing amounted to 39 ms (SE = 15.2), which was significant, *F*_(1,17)_ = 6.37, *p* = 0.022, *η*_p_^2^ = 0.272. However, the ANOVA on RT did not reveal any further effects, all *p*s < 0.447, *η*_p_^2^ = 0.090. Thus, post-error increase of accuracy was reduced in the rACC group as compared to the other two groups. By contrast, post-error slowing did not differ between the groups.

## Discussion

The present study tested whether impairments of rapid outcome evaluation as measured by ERPs lead to impairments of error awareness. The results show that the difference between errors and correct responses (i.e., the ΔNe/ERN, see also, e.g., Gehring and Knight, 2000; Yeung et al., 2004; Turken and Swick, 2008; Steinhauser and Yeung, 2010; Wessel et al., 2014) was strongly attenuated in a group of patients with lesions of the rACC, while large ΔNe/ERNs were observed in age-matched healthy controls and patients with lesions outside the frontal lobes. Nevertheless, rACC patients signaled their errors equally well as controls. This demonstrates that damage to brain systems, which are crucial for the ΔNe/ERN can leave the later emergence of error awareness unaffected.

These results have important implications for theories of error monitoring. The Ne/ERN was previously found to be diminished by lesions in various areas of fronto-striatal circuits. For instance, lesions of the dACC (Swick and Turken, 2002), the dorsolateral PFC (DLPFC; Gehring and Knight, 2000; Ullsperger et al., 2002; Ullsperger and von Cramon, 2006; Wessel et al., 2014), the thalamus (Seifert et al., 2011), frontal white matter (Hogan et al., 2006), and basal ganglia (Ullsperger and von Cramon, 2006) were all shown to diminish the Ne/ERN. By contrast, error awareness was found to be impaired by lesions of the DLPFC (Hoerold et al., 2013), and the thalamus (Seifert et al., 2011), but spared by lesions of both the dACC and the rACC (Modirrousta and Fellows, 2008). The present findings of attenuated ΔNe/ERN amplitudes in the presence of intact error awareness in patients with lesions of the rACC add to this literature by showing that an intact ΔNe/ERN is not a necessary precondition for error awareness to emerge.

Thus, error awareness can arise also when the neural system generating the ΔNe/ERN is damaged, and the ΔNe/ERN therefore does not seem to provide critical information for error awareness. This raises the question, of how error awareness is otherwise achieved. A recent mechanistic account of error detection (Steinhauser et al., 2008) holds that a tendency for internal self-corrections forms the basis for error awareness (see also, Rabbitt et al., 1978; Rabbitt and Vyas, 1981). Specifically, this response monitoring account of error detection assumes that error detection is achieved by a mechanism which compares the initially executed response with the response activated during extended processing of the stimulus after error commission and, if it detects a discrepancy, it concludes that the first response was an error. Indeed, manipulating the response criterion affects error signaling responses and overt error correction responses in a highly similar way (Steinhauser et al., 2008; see also Steinhauser and Yeung, 2012) supporting the idea that error signaling is based on internal error corrections. In the present study, not only the frequency of error signaling responses, but also the frequency of spontaneous error corrections was entirely intact in rACC patients (see also, Modirrousta and Fellows, 2008). As the frequency of spontaneous error corrections can be regarded as an estimator for internal error correction activity (e.g., Rabbitt, 2002), this is in accordance with the notion that error detection is achieved by monitoring internal error corrections.

Also if the ΔNe/ERN is not necessary for error awareness as suggested by the present results, it is conceivable that both error awareness and the ΔNe/ERN rely on a common process further upstream in the error monitoring system. For instance, in line with the fact that lesions of the DLPFC lead to impairments of both error awareness (Hoerold et al., 2013) and the Ne/ERN (Gehring and Knight, 2000; Ullsperger et al., 2002; Ullsperger and von Cramon, 2006; Wessel et al., 2014), the DLPFC could provide information, which is necessary for both processes. Internal error correction activity seems a good candidate for such a source of information. Error corrections are often impaired in patients with lesions of the DLPFC (Gehring and Knight, 2000; Ullsperger and von Cramon, 2006; Wessel et al., 2014). Interestingly, the conflict monitoring account of the Ne/ERN (Botvinick et al., 2001; Yeung et al., 2004), assumes that post-error response conflict between the executed response and the self-correction tendency gives rise to the Ne/ERN. Thus, internal error correction mediated by the DLPFC could provide the basis for both error awareness as suggested by the response monitoring account (Steinhauser et al., 2008), and the generation of the Ne/ERN in the ACC as suggested by the conflict monitoring account (Yeung et al., 2004).

The present results also have implications for the functional role of the ΔNe/ERN in error monitoring. rACC patients not only displayed reduced ΔNe/ERN amplitudes, but also failed to increase response accuracy on trials following errors. Notably, rACC patients showed preserved post-error slowing. This shows that post-error slowing and post-error increase of accuracy do not necessarily co-occur (see also, Danielmeier and Ullsperger, 2011, for a review) and that post-error slowing *per se* is not necessarily adaptive. In accordance with previous correlational evidence of a relationship between rapid error monitoring and adaptive post-error behavioral adjustments (Gehring et al., 1993; Ridderinkhof et al., 2002; Debener et al., 2005; Marco-Pellarés et al., 2008; Carp and Compton, 2009; Maier et al., 2011; Themanson et al., 2012), this suggests a causal role of the ΔNe/ERN for adjusting behavior following errors. Such a causal role is also in line with current theoretical accounts of the Ne/ERN. In addition to the already mentioned conflict monitoring account of the Ne/ERN (Botvinick et al., 2001; Yeung et al., 2004), other mechanisms such as detection of a mismatch between the intended and the actual response (Falkenstein et al., 2000; Coles et al., 2001) or detection of violations of outcome predictions (Holroyd and Coles, 2002; Alexander and Brown, 2011) were also assumed to underlie the generation of the Ne/ERN. Notably, each of these mechanisms is principally suited to signal the need for adjusting behavior. Post-error response conflict, mismatch between actual and intended response, and violations of outcome predictions all imply that the performance goal was not met and behavioral adjustments are necessary to optimize performance. Therefore, all mentioned accounts predict a relationship between Ne/ERN amplitudes and adaptive post-error behavioral adjustments (see, Ridderinkhof et al., 2004), in accordance with the present results.

However, our results also suggest that existing theories of the Ne/ERN should be extended. Namely, we show that not only dorsal regions of the ACC (Ridderinkhof et al., 2004), but also the more rostrally located rACC and adjoining ventromedial PFC are involved in rapid outcome evaluation and adaptive post-error behavioral adjustments (see also, di Pellegrino et al., 2007; Turken and Swick, 2008). These regions are often implicated in the regulation of behavior in affective contexts (Etkin et al., 2006; Egner et al., 2008; Maier and di Pellegrino, 2012). Our results show that although these regions more associated with the affective system are not necessary for conscious error awareness, they are crucial for rapid error monitoring and post-error behavioral adjustments. Such an involvement of affective aspects in rapid outcome evaluation (Luu et al., 2000; Hajcak and Foti, 2008) is in accordance with evidence from functional imaging suggesting that while errors in general activate the dACC, errors on incongruent trials additionally produce enhanced activity in the rACC (Wittfoth et al., 2008). As errors on incongruent trials often imply failures to ignore the distractor dimension of the stimulus and are hence particularly significant for optimizing performance (Maier et al., 2012), an enhanced recruitment of the rACC on these trials might indicate a stronger involvement of the affective system for these errors. This interpretation receives support from a close association of rACC activity and error-induced changes in autonomic arousal (Critchley et al., 2005). Thus, together with the existing literature, our results suggest a more heterogeneous multi-process rapid outcome evaluation system with different but not mutually exclusive mechanisms that work in concert to signal the need for adaptive post-error behavioral adjustments according to the task demands.

Our findings also allow for some speculation on a possible architecture of the error monitoring system. Namely, we found that the reduction of the ΔNe/ERN in rACC patients was more due to an enhancement of the negativity in the Ne/ERN time window on correct trials than due to a reduction of the negativity on error trials. This finding closely resembles the pattern of results obtained in an earlier study involving patients with lesions of the dLPFC (Gehring and Knight, 2000). These authors proposed that the dACC generates the Ne/ERN, but in so doing depends on information from the DLPFC about contextually appropriate stimulus-response mappings to distinguish between correct and error responses. Without such information from the DLPFC, the dACC would by default produce a Ne/ERN explaining the similar negativities on correct and error trials in patients with DLPFC damage. A similar scenario is also conceivable with rACC damage: in accordance with its presumed role in emotional processing, the rACC would provide the dACC with information about the emotional significance of errors. Without such information from the rACC, the dACC would by default generate a negativity in the peri-response window leading to similar negativities on correct and error trials. Importantly, such a scenario would also be in accordance with the notion that the system triggering the Ne/ERN does not provide the crucial information for later error awareness. If a negativity in the peri-response window *per se* led to error awareness, then one would expect an increase in the false alarm rate (i.e., rACC patients would have more often falsely signaled errors on correct trials), which we did not observe.

Finally, the preserved ability of rACC patients to signal their errors went along with an intact Pe component. An intact Pe together with preserved error signaling is in accordance with previous evidence that the Pe rather than the Ne/ERN is associated with error awareness (Nieuwenhuis et al., 2001; Endrass et al., 2005, 2007, 2012; O’Connell et al., 2007; Steinhauser and Yeung, 2010, 2012) and thus, with the view that Ne/ERN and Pe reflect dissociable neural processes (see also, Scheffers and Coles, 2000; Overbeek et al., 2005; Ridderinkhof et al., 2009; Hewig et al., 2011; Hughes and Yeung, 2011). Notably, the Pe seemed to occur somewhat later in rACC patients than in healthy controls. However, this difference was not significant. Moreover, Pe latencies were very similar between rACC patients and BDC patients suggesting that a possible delay in the Pe is not specific for rACC lesions, but rather is a result of generally slowed processing in brain damaged individuals.

Although the present study has the advantage of including a brain-damaged control group in addition to a group of neurologically healthy controls, a limitation of the present study is the small sample size. Given that patient studies like the present one are relatively rare, the present data should be regarded as adding to the existing evidence on the effects of lesions in the ventromedial prefrontal cortex on error monitoring processes.

In sum, the present study showed that error awareness can arise even if rapid error monitoring is severely attenuated due to lesions of the rACC, which demonstrates that rapid error monitoring and error awareness are dissociable. Furthermore, reduced ΔNe/ERN amplitudes coincided with a failure to adaptively adjust behavior following errors, which supports the notion that associated rapid error monitoring processes signal the need for adaptive post-error behavioral adjustments. Finally, intact Pe amplitudes in the presence of intact error awareness and concurrently diminished ΔNe/ERN amplitudes is in line with the idea that the Pe is linked to the emergence of error awareness and is dissociable from rapid error monitoring processes.

## Author Contributions

MEM, GDP, FDG and TM designed the experiments. MEM, FDG and TM recruited participants and collected the data. MEM and GDP performed the lesion reconstructions. MEM and FDG performed the data analyses. MEM and GDP wrote the manuscript.

## Conflict of Interest Statement

The authors declare that the research was conducted in the absence of any commercial or financial relationships that could be construed as a potential conflict of interest.
